# Relationship between Sleep Bruxism, Perceived Stress, and Coping Strategies

**DOI:** 10.3390/ijerph16173193

**Published:** 2019-09-01

**Authors:** Klara Saczuk, Barbara Lapinska, Paulina Wilmont, Lukasz Pawlak, Monika Lukomska-Szymanska

**Affiliations:** Department of General Dentistry, Medical University of Lodz, 251 Pomorska St., 92-213 Lodz, Poland

**Keywords:** sleep bruxism, perceived stress, coping strategies

## Abstract

Sleep bruxism (SB) is a common phenomenon defined as a masticatory muscle activity during sleep. Untreated severe SB can have significant dental and orofacial consequences. SB has often been linked with stress and maladaptive coping strategies. Therefore, in this study, a potential correlation between SB, perceived stress and coping strategies was evaluated. A total of 60 adults were enrolled into this study. Participants underwent a detailed intra- and extraoral exam focused on detecting bruxism symptoms. Additionally, the overnight Bruxism Index was recorded using the Bruxoff device. A total of 35 participants with symptoms of bruxism were assigned to the study group, whereas 25 asymptomatic participants were assigned to the control group. The Perceived Stress Scale (PSS-10) was used for stress assessment and Brief-COPE for coping strategies. Results showed that the higher the PSS-10 score, the higher the Bruxism Index was in the study group. Positive coping strategies were chosen most frequently in the control group, while maladaptive ones were chosen in the study group. It can be concluded that there is a relationship between perceived stress and sleep bruxism. Moreover, the type of coping strategies used by participants may have an impact on sleep bruxism, but the relationship should be further investigated.

## 1. Introduction

Sleep bruxism (SB) is becoming a common phenomenon, affecting 8–31% of the adult population, irrespective of gender. The prevalence of SB is reported to be decreasing with age [1]. According to the International Classification of Sleep Disorders, bruxism is described as a “repetitive jaw-muscle activity characterized by clenching or grinding of the teeth and/or by bracing or thrusting of the mandible” [2]. However, a new definition from the 2018 International Consensus [3] defines sleep bruxism as “a masticatory muscle activity during sleep that is characterized as rhythmic (phasic) or non-rhythmic (tonic) and is not a movement disorder or a sleep disorder in otherwise healthy individuals”. The Consensus also points out that sleep bruxism should be regarded as a different behavior than awake bruxism. Despite the existence of positive aspects of SB such as allowing unobstructed airway passage while sleeping and aiding salivation [4], severe bruxism has mainly destructive consequences e.g., muscle hypertrophy and/or pain, temporomandibular disorders, loss of periodontal support, dental implants and tooth structure [5,6,7].

SB etiology is still complex. Occlusal theories linking SB with occlusal and anatomical disorders have not gained sufficient supporting evidence [8,9,10], but there is much research confirming a significant connection between the rhythmic (phasic) masticatory muscle activity (RMMA) observed in SB and an increased activity from the central nervous system. It has been observed that most SB episodes occur along both brain and cardiac reactivations (“micro-arousals”) and therefore, it is believed that SB can be closely related to the central nervous system (both sympathetic and parasympathetic) [11,12]. Psychological factors also seem to play an important role in SB etiology. Stress, which has long been linked to health issues, both mental and physical [13,14,15] could be associated with SB since levels of salivary cortisol and catecholamines, indicating stressful states, were raised among bruxers [16,17,18,19]. Other research disputes the role of stress [20], but underlines anxiety as a valid aspect of SB [21,22,23,24]. Coping mechanisms, despite being rarely investigated in relation to SB, have also gained much recognition as possible aspects of the etiology of SB and could have an important role as causative factors [25,26]. Taking into account the above-mentioned research on SB etiology and stress being called the “Health Epidemic of the 21st Century” by the World Health Organization [27], the relationship between SB and psychological aspects seems to be worth further investigation.

The aim of this paper was to evaluate the relationship between perceived stress, coping mechanisms and sleep bruxism. The null hypotheses of this paper were: (1) there is no correlation between sleep bruxism and perceived stress; (2) there is a no correlation between sleep bruxism and coping strategies; (3) there is no correlation between gender and coping strategies.

## 2. Materials and Methods

### 2.1. Study Design

The study included 60 adult and consenting participants; 35 for the study group and 25 for the control group. Participants were enrolled between June 2017 and March 2019. The inclusion criteria for the study and control groups were: willingness to participate in the study, between 18 and 66 years old, male or female, and in generally good health. The exclusion criteria for both groups were: unwillingness to participate in the study, severe mental illness, severe neuromuscular illness, undergoing prosthodontic rehabilitation, more than two missing teeth per quadrant (excluding third molars) [28], under medication which impaired neuromuscular system activity, under antipsychotic or antidepressant drugs, any severe chronic illness, cognitive disability, active inflammation, present oncologic condition and smoking.

Each participant underwent an intraoral and extraoral examination performed by the same trained dentist. The examination was focused on finding bruxism symptoms in the oral cavity: indentation on the tongue and/or linea alba on the inner cheek, damage to the dental hard tissues or mechanical wear of the teeth and a muscle palpation test. Masseter and temporal muscles were examined, both the origins and heads of the muscles, for signs of pain. In addition, the study used a portable electromyography/electrocardiogram (EMG/ECG) device (Bruxoff, Bioelettronica, Turin, Italy), according to the protocol described by Saczuk et al. [29], to evaluate the overnight Bruxism Index (number of bruxism episodes per hour) in both groups. Based on the examination, participants were divided into study and control groups. In the study group, all participants were diagnosed with clinical bruxism symptoms in the oral cavity, self-reported bruxism and a Bruxism Index higher than 2. Consequently, none of the participants in the control group exhibited bruxism symptoms (including muscle pain), self-reported bruxism or a Bruxism Index higher than 2.

For stress assessment, the authors used the Perceived Stress Scale (PSS-10), a self-report tool composed of 10 statements regarding “how unpredictable, uncontrollable, and overloaded respondents find their lives” [30]. Each item on the PSS-10 ranges from 0 (never) to 4 (very often) on a 5-point Likert scale. The PSS-10 consists of 6 positive statements (1, 2, 3, 6, 9 and 10) and 4 negative statements (4, 5, 7 and 8). During the evaluation, negative statements are re-coded and the scores are calculated. Total scores range from 0 to 40 and the higher the score, the higher perceived stress levels [30]. PSS-10 scores ranging 0–13 were considered low perceived stress, 14–19 were considered moderate perceived stress and 20–40 were considered high perceived stress [31]. Since all participants were of Polish nationality, the PSS-10 results were converted to the sten scale (standard ten) [31]. The sten scale is the scale of the psychological test normalized so that the average in the population is 5.5 sten, and the standard deviation is 2. Therefore, PSS-10 scores ranging from 0–13 (1–4 sten) were considered low perceived stress, 14–19 (5–6 sten) were considered moderate perceived stress and 20–40 (7–10 sten) were considered high perceived stress [31].

For coping mechanisms the authors used the Brief-COPE scale [32], a self-report tool composed of 28 statements regarding 14 strategies of coping with stress. Each one of the 14 strategies (Active Coping, Planning, Positive Reframing, Acceptance, Humor, Seeking Emotional Support, Seeking Instrumental Support, Self-Distraction, Denial, Venting, Substance Use, Behavioral Disengagement, Self-Blaming) corresponds with two particular statements of the 28 in the Brief-COPE. Each item on the Brief-COPE ranges from 0 (almost never) to 3 (almost always). During the evaluation, scores for each coping strategy are calculated by adding together the points from two corresponding statements and then dividing it by two. Total scores range from 0 to 3 for each coping strategy and the higher the score for a particular strategy, the more preferred it is by the participant.

The study was approved by the Local Ethical Committee (RNN/173/17/KE). All participants signed the consent form prior to inclusion in the experiment.

### 2.2. Statistical Analysis

For qualitative variables, the structure indices were calculated and expressed in %. The following characteristics were calculated for measurable variables: arithmetic mean (x) and median (Me) as average values, and standard deviation (SD) and the coefficient of variation (CV) as measures of dispersion. The test value (z) and minimum (min) and maximum (max) values were also given. The normality of the quantitative variable distributions was checked using the Shapiro-Wilk test prior to comparison of the averages. As the distributions of variables significantly differed from the normal distribution, the non-parametric Mann-Whitney test was used to compare means in two groups (study and control). In the study of the relationship between measurable variables, the rank correlation coefficient was calculated. A level of *p* < 0.05 was considered statistically significant. If theoretical numbers were less than 5, then the Yates amendment was taken into account, and if smaller than 3, Fisher’s exact test was used and there was no chi-square, only *p*.

## 3. Results

In the study group, 12 males and 23 females were enrolled, aged 20 to 56 years old with an average age of 29.9 years ± 8.35. In the control group, 5 males and 20 females were enrolled, aged 22 to 66 years old with an average age of 35 ± 10.9.

In the study group, a positive correlation (medium power) [33] between the PSS-10 score and the Bruxism Index was found (rank correlation coefficient was 0.530, *p* < 0.01) (Table 1). In other words, the higher the PSS-10 score, the higher the Bruxism Index. In contrast, in the control group there was no such statistically significant relationship (*p* > 0.05).

Comparison of the average PSS-10 results between the study and the control group showed that the results in the study group were significantly higher than in the control group (*p* < 0.05) (Table 2).

There was a statistically significant difference between the study and the control group in the frequency of PSS-10 results: low, moderate and high (*p* < 0.05) (Table 3). A detailed comparison of the results showed that the study group had significantly higher results (28.6% vs. 4.0%) than the control group, whereas in the control group low and moderate results significantly predominated (40.0% vs. 34.3% and 56.0% vs. 37.1%, respectively).

There was no statistically significant difference between average PSS-10 results in men and women in both groups (*p* > 0.05) (Table 4).

Also, in the study group as well as in the control group, there was no statistically significant difference between persons of both sexes in the frequency of PSS-10 results: low, moderate and high (*p* > 0.05) (Table 5).

The comparison of the frequency of selected ways of coping with stress (Table 6), showed that the significantly most often selected coping strategy in the study group was self-distraction (34.4%, *p* < 0.01), whereas in the control group it was planning (44.0%, *p* < 0.01). When comparing the frequency of the most often selected coping strategies between the groups, it turned out that subjects from the study group chose self-distraction (34.3% vs. 0.0%, *p* < 0.01) and self-blaming (25.7% vs. 0.0%, *p* < 0.05) significantly more often than the control group.

As for the control group, planning (44.0% vs. 8.6%, *p* < 0.01), acceptance (20.0% vs. 0.0%, *p* < 0.05) and turning towards religion (24.0% vs. 2.9%, *p* < 0.05) were chosen significantly more often than in the study group.

Among men of the study group, the most often chosen coping strategy was self-distraction (50.0%), while women chose self-blaming (30.4%). A comparison of men and women from the study group in terms of the frequency of choosing different ways of coping with stress showed a statistically significant difference. Men more often than women sought emotional support (33.3% vs. 4.4%, *p* < 0.05) (Table 7).

A comparison of men and women from the control group in terms of the frequency of choosing different ways of coping with stress did not show any statistically significant difference (*p* > 0.05) (Table 7). For both genders, active coping and planning were the most often chosen coping strategies. Women also tended to seek emotional support.

## 4. Discussion

To evaluate perceived stress, the PSS-10 was chosen from among three existing PSS versions. PSS-10 was regarded as reliable, valid and of psychometric properties superior to those of both 14-PSS and 4-PSS [34]. The present study showed the statistically significant correlation between the PSS-10 score and Bruxism Index, evaluated using the Bruxoff device. According to previous studies, Bruxoff sensitivity is estimated at 92–100%, whereas its specificity ranges from 76% to 91.6% [29,35,36], showing high reliability of the device in diagnosing bruxism. The results of the present study emphasized the notion that the higher perceived stress score, the higher the Bruxism Index. A detailed comparison of the results showed that the participants from the study group experienced significantly higher perceived stress than the participants from the control group. This finding seemed to confirm the overall view that stress could be correlated with sleep bruxism [26,37]. Other studies have analyzed the association between bruxism and stress experience, age, gender, work role and occupational health care [38]. It was found that perceived stress correlated with SB regardless of specified type of work and that frequent bruxers reported more stress. Later, in a follow-up study to the above-mentioned paper, it was again confirmed that the role of stress as a factor in sleep bruxism should not be ignored [39]. Chronic stress and associated bodily reactions could be responsible for both muscle tension, including in the masticatory muscles, and pain. These hallmarks of SB may indicate that stress plays a paramount role in the development of SB [40]. Additionally, those stress-triggered responses could in turn lead to several neuromuscular disorders [41]. Furthermore, individuals with a tendency to be controlling, compulsive and tending towards aggressive behavior are more prone to developing bruxism [42]. However, in light of other papers disproving the stress-SB correlation, the subject needs further study [43,44,45].

When evaluating the overnight Bruxism Index in subjects, sleep length could not be evaluated due to the lack of electroencephalograph (EEG) electrodes in the Bruxoff device. Since SB is proved to be correlated not only with stress, but also with anxiety, which is said to correspond with sleep disorders like insomnia [46,47], it could be worth further study.

The study evaluated the frequency of low, moderate and high PSS-10 scores in both groups. The comparison of those results showed a statistically significant difference. Even though average scores dominated in both groups, the study group had statistically significantly fewer low results and statistically significantly higher results than the participants from the control group. That outcome correlates with the fact that the study group experienced higher perceived stress and in turn, had a higher overnight Bruxism Index, which further confirms the correlation between stress and SB.

In the present study, there was no statistically significant difference within the study group and the control between genders in the frequency of low, moderate and high PSS-10 results. However, the low number of participants and lack of gender parity could have been influencing factors. In addition, this finding is in contrast with some research claiming that stress-related disorders have a higher incidence in women than in men [48,49]. Other studies have revealed differences between genders on both the molecular and whole systems levels, which can be responsible for the increase in endocrine, emotional, and arousal responses to stress in females [50]. However, women have also been shown to handle stress better, by being less susceptible to the memory-impairing effects of elevated cortisol levels than men [51]. In terms of highly-ranking work-related stress, higher results in women were due to women being more stressed by their “greater unpaid workload and by a greater responsibility for duties related to home and family” [52].

In terms of coping strategies, the Polish-adapted version of Brief-COPE was chosen due to its confirmed assumption that an individual’s coping strategies remain relatively consistent across various types of stressors [53]. Additionally, according to the 2013 meta-analysis of coping scales, COPE and its short or revised versions were the most frequently used in research papers [53]. The same meta-analysis also showed that COPE was the most frequently used scale in papers involving participants.

Based on the Brief-COPE questionnaire results, the study showed that participants in the study group most frequently chose self-distraction, followed by self-blaming, which are said to be correlated with a low level of well-being [53]. These results confirmed previous studies, reporting that self-distraction was associated with high SB sensitivity and greater masseter muscle activity, and was also correlated with avoidant personality characteristics [54]. None of the people from the study group adopted positive reframing and acceptance, which were associated with adversarial growth [55]. In the control group, planning, acceptance and turning towards religion were selected significantly more frequently compared to the study group. This showed that participants with sleep bruxism more commonly chose maladaptive coping strategies.

Positive coping strategies, which are said to reduce stress and are correlated with a high level of well-being [53], prevailed among participants in the control group and were less visible in the study group. Furthermore, none of the respondents from the control group chose self-distraction, denying, venting, substance use or self-blaming, which are said to be forms of avoidance coping [56]. This also confirmed observations made in other studies, which reported a deficit of functional coping strategies in participants with sleep bruxism [26,57].

Comparison of men and women from the study group in terms of coping with stress showed that men preferred to seek emotional support significantly more often than women. However, other research showed emotional coping as being predominantly chosen by females [58]. Despite the lack of statistical significance, it is also worth noting that men chose self-distraction more than women, whereas women more often opted for blaming themselves. Previously published results indicated that men engaged in significantly more use of humor, whereas women utilized significantly more emotional and instrumental support to cope with stress [59]. The same study revealed that the coping strategies of denial and self-blame significantly predicted perceived stress for women, while behavioral disengagement and self-blame were significant predictors for men. In the control group, no statistically significant difference was found between men and women in the frequency of choosing stress coping strategies.

Personality types were not assessed in this study. However due to personality traits’ influence on stress responses, anxiety and coping strategies, they could have been important factors [60,61,62]. Correlations between PSS-10 scores, Brief-COPE score and age were not investigated in this study due to a wide age range in the study and control group, and a relatively low number of participants. All of the above-mentioned, along with the lack of gender parity within both groups and lack of EEG electrodes are considered the limitations of the present study.

## 5. Conclusions

Within the limitations of the present study, it can be concluded that there is a relationship between perceived stress and sleep bruxism. It could also be concluded that the type of coping strategies used by participants may have an impact on sleep bruxism, but the relationship should be further investigated. Moreover, there is a correlation between gender and preferred coping strategy, but the subject demands in-depth research.

## Figures and Tables

**Table 1:** Correlation between Perceived Stress Scale (PSS-10) result and Bruxism Index in the study and control groups.

	Group	
	Study	Control
**Rank Correlation**	0.530	0.167
**Test Value t**	3.594	0.814
Significance *p*-Value *	**0.0010**	0.424

* statistically significant *p* values were bolded.

**Table 2:** Comparison of PSS-10 results in the study and control groups.

Group	Calculated PSS-10 Parameters					
	Min	Max	X	Me	SD	V (%)
**Study**	9	32	18.6	18.0	6.74	36.2
**Control**	5	23	14.6	15.0	5.36	36.6
Comparison *	z = 2.047; ***p* = 0.0406**					

* statistically significant *p* values were bolded.

**Table 3:** Comparison of the frequency of low, moderate and high PSS-10 results in the study and control groups.

PSS-10 Result	Group			
	Study		Control	
	*n*	%	*n*	%
Low	12	34.3	10	40.0
Moderate	13	37.1	14	56.0
High	10	28.6	1	4.0
Total	35	100.0	25	100.0
Comparison *	chi^2^ = 7.897; ***p* = 0.0193**			

* statistically significant *p* values were bolded.

**Table 4:** Comparison of PSS-10 results in men and women in the study and control groups.

Gender	Calculated PSS-10 Parameters					
	Min	Max	X	Me	SD	V (%)
	**Study Group**					
**Men**	12	29	19.4	18.5	6.21	32.0
**Women**	9	32	18.2	16.0	7.10	39.0
**Comparison**	z = 0.643; *p* = 0.520					
	**Control Group**					
**Men**	10	20	14.6	14.0	4.77	32.7
**Women**	5	23	14.7	15.5	5.61	38.3
**Comparison**	z = 0.102; *p* = 0.919					

**Table 5:** Comparison of the frequency of low, moderate and high PSS-10 results in men and women in the study group.

	Study Group					Control Group				
PSS-10 Result	Gender				Total	Gender				Total
	Men		Women			Men		Women		
	*n*	%	*n*	%		*n*	%	*n*	%	
Low	3	25.0	7	30.4	10	2	40.0	8	40.0	10
Moderate	4	33.3	9	39.2	13	3	60.0	11	55.0	14
High	5	41.7	7	30.4	12	-	-	1	5.0	1
Total	12	100.0	23	100.0	35	5	100.0	20	100.0	25
	chi^2^ = 0.443; *p* = 0.801					chi^2^ = 0.268; *p* = 0.875				

**Table 6:** Comparison of the frequency of selected methods of coping with stress by persons from the study and control groups.

Brief-COPE	Group				Chi^2^	*p*-Value *
	Study		Control			
	*n*	%	*n*	%		
**Active coping**	7	20.0	10	40.0	2.871	0.090
**Planning**	3	8.6	11	44.0	10.230	**0.0014**
**Positive Reframing**	-	-	4	16.0	3.713	0.054
**Acceptance**	-	-	5	20.0	5.246	**0.0220**
**Humor**	3	8.6	3	12.0	0.016	0.898
**Religion**	1	2.9	6	24.0	4.442	**0.0351**
**Emotional Support**	5	14.3	7	28.0	1.718	0.190
**Instrumental Support**	3	8.6	3	12.0	0.000	1.000
**Self-distraction**	12	34.3	-	-	10.710	**0.0011**
**Denial**	2	5.7	-	-	-	0.506
**Venting**	4	11.4	-	-	-	0.133
**Substance use**	1	2.9	-	-	-	0.583
**Behavioral Disengagement**	7	20.0	1	4.0	1.993	0.158
**Self-blaming**	9	25.7	-	-	5.676	**0.0172**

* statistically significant *p* values were bolded.

**Table 7:** Comparison of the frequency of selected methods of coping with stress by men and women from the study and the control group.

Brief-COPE	Study Group Gender				Chi^2^	*p*-Value *	Control Group Gender				Chi^2^	*p*-Value *
	Men		Women				Men		Women			
	*n*	%	*n*	%			*n*	%	*n*	%		
Active coping	2	16.7	5	21.7	0.008	0.929	3	60.0	7	35.0	0.260	0.610
Planning	-	-	3	13.0	-	0.536	3	60.0	8	40.0	0.091	0.763
Positive Reframing	-	-	-	-	-	-	-	-	4	20.0	-	0.549
Acceptance	-	-	-	-	-	-	1	20.0	4	20.0	-	0.708
Humor	2	16.7	1	4.4	-	0.266	1	20.0	2	10.0	-	0.504
Religion	-	-	1	4.4	-	0.657	1	20.0	5	25.0	-	0.657
Emotional Support	4	33.3	1	4.4	-	0.0375	-	-	7	35.0	-	0.274
Instrumental Support	1	8.3	2	8.7	-	0.735	1	20.0	2	10.0	-	0.504
Self-Distraction	6	50.0	6	26.1	2.003	0.157	-	-	-	-	-	-
Denial	-	-	2	8.7	-	0.536	-	-	-	-	-	-
Venting	1	8.3	3	13.0	-	0.575	-	-	-	-	-	-
Substance use	-	-	1	4.4	-	0.657	-	-	-	-	-	-
Behavioral Disengagement	2	16.7	5	21.7	0.008	0.929	-	-	1	5.0	-	0.800
Self-blaming	2	16.7	7	30.4	0.228	0.633	-	-	-	-	-	-

* statistically significant *p* values were bolded.
